# Liquid Biopsy, an Everchanging Balance between Clinical Utility and Emerging Technologies

**DOI:** 10.3390/cancers14174277

**Published:** 2022-09-01

**Authors:** Linda Cucciniello, Lorenzo Gerratana, Fabio Puglisi

**Affiliations:** 1 Department of Medicine (DAME), University of Udine, 33100 Udine, Italy; 2 Department of Medical Oncology, Centro di Riferimento Oncologico di Aviano (CRO), IRCCS, 33081 Aviano, Italy

To date, tissue biopsy still represents the mainstay for tumor diagnosis and molecular characterization. However, it comes with several disadvantages, including the difficulty in retrieving adequate tumor samples and performing longitudinal monitoring, together with its inability to fully grasp both tumor heterogeneity and biological evolution [1,2,3]. The aim of this Special Issue is to describe how liquid biopsy can serve in clinical practice, as well as the advances in the development of novel liquid biopsy technologies.

Liquid biopsy is a minimally invasive procedure that has gained momentum due to its capacity to overcome the aforementioned limitations. Blood is the most common matrix, but alternatives such as urine or cerebrospinal fluid can be used. Tumor cells are continuously releasing components within body fluids, such as nucleic acids, including DNA (cell-free DNA (cfDNA) and circulating tumor DNA ctDNA) and microRNAs (miRNAs), but also vesicles and proteins. This process occurs when cancer cells spread in the organism as single units (circulating tumor cells, CTCs) or as clusters (CTC-c), a phenomenon not only confined to late stages [4,5].

By analyzing such components, [especially cfDNA and cell-free RNA (cfRNA)] it is possible to evaluate the response to a specific treatment, identify potential mechanisms of resistance and, in perspective, modify the treatment accordingly [6,7,8,9,10]. However, only a limited amount of ctDNA is obtainable from peripheral blood, and thus, a proper preanalytical workflow is crucial [11,12,13]. In particular, through the simultaneous analysis of cfDNA and cfRNA in the serial plasma samples, a direct comparison can be performed to assess how the disease burden changes over time and understand how it is responding to the treatments [13].

Moreover, changes in disease biology and the onset of mechanisms of resistance can derive not only from genomic-based alterations, but also from epigenetic and transcriptomics changes that can take place in the early stages of the disease [14,15]. Such events can be described through the use of several liquid biopsy tools and can facilitate both disease diagnosis and treatment decision making [10,16,17]. In particular, DNA methylation patterns are organ specific and appear to be feasible biomarkers to support diagnostic imaging [15,17]. As a matter of fact, in metastatic colorectal cancer (mCRC), a RAS mutation can become undetectable due to disease progression or because of a decrease in ctDNA shedding. In this scenario, the use of a specific methylation panel was found to be helpful in guiding the differential diagnosis. This finding is of great clinical importance, since patients with mCRC switching to a RAS wild-type phenotype could be candidate for an anti-EGFR-based therapy at progression [18].

Liquid-biopsy-based clinical diagnostics have gained an increasing importance in recent years, alongside the emergence of targeted therapies.

Approximately 15% of advanced NSCLC present with activating mutations in *EGFR*, which can be targeted by tyrosine kinase inhibitors (TKI) [19]. Liquid biopsy can be employed not only to determine the presence of activating *EGFR* mutations in treatment-naïve patients for which the tissue sample is insufficient or inadequate for the molecular analysis, but also in patients with a known *EGFR* mutation, after disease progression to first- or second-generation TKIs, to identify the T790M resistance mechanism [20]. The identification of *EGFR* mutations is also crucial for the management of early stage NSCLC patients that can be candidates for adjuvant therapy with the 3° generation TKI Osimertinib.

The search of cell-derived elements through liquid biopsy can also help to identify cases of disease recurrence, as shown for sarcomas presenting with *EWSR1*-associated translocations [21]. This is a field of research that is also being explored in early stage breast cancer (eBC) and early stage CRC (eCRC).

Aside from ctDNA, miRNAs are also promising blood-based biomarkers. Circulating miRNA signatures have been found in the plasma of patients with lung cancer, and a three-miRNA signature (mi-R-16-5p, miR-92a-3p and miR-451a) has been found to be associated with the modulation of major signal transduction pathways in lung cancer, including *EGFR*, *K-RAS* and *PI3K/AKT* [22].

Extracellular vesicles (EVs) are membrane-bound nanoparticles containing proteins and nucleic acids, including mRNA, miRNA and non-coding RNA. A recently published study investigated the potential use of a targeted RNA sequencing panel to evaluate tissue and plasma samples from glioblastoma multiforme (GBM) for RNA fusion transcripts. The fusion transcript FGFR3-TACC3 was detected in both tissue and plasma samples, and its longitudinal evaluation suggested the feasibility of plasma-based RNA fusion monitoring [23]. EVs can, moreover, remodel the tumor immune microenvironment (TME) and modulate the crosstalk among cancer cells, immune cells and other cells encompassing the TME. Analyzing the elements enclosed in circulating EVs is a promising strategy to better understand the dynamics of tumor immune regulation, and it could serve as a tool for liquid biopsy in cancer immunotherapy. In NSCLC, for example, the reduced presence of endothelial-derived EVs in blood is related to response to immune checkpoint inhibitors [24]. Similar to other cancer-derived elements, EVs can be found not only in peripheral blood, but also in other matrices. For example, urinary-derived EVs and urinary bladder cancer (UBC) organoids (i.e., ex vivo mini tumors grown from a patient’s tumor fragment) have been found to be predictive biomarkers of response to chemotherapy [25,26].

CTCs and CTC-c have been regarded as the potential unit for metastatic spreading [27,28]. CTC-c are extremely rare, representing a challenge for their deployment as liquid biopsy and prompting for the development of detection systems with greater sensitivity and specificity. The CellSearch system is the gold standard for the enumeration of CTCs in a standardized and validated manner [29]. However, due to its dependency on the epithelial cell adhesion molecule (EpCAM), this system can identify only cancer cells with epithelial phenotypes [29].

However, metastatic spreading is related to epithelial-to-mesenchymal transition, and mesenchymal CTCs are more invasive, chemo resistant and have been associated with reduced survival outcome.

Consequently, the development of non-EpCAM-based assays capable of identifying also mesenchymal CTCs represents a major challenge. In this regard, alternative methods capable of extracting CTCs from peripheral blood based upon their size and deformability with respect to leukocytes have been successfully evaluated. Supporting evidence also comes from another study in which the use of a size-based tool enabled the detection of CTC-c in patients with eBC, as well as in cases classified as CTC-c negative by CellSearch [4,5].

CTC-c are more frequently observed in eBC rather than in MBC, thus sustaining the hypothesis that cancer cell dissemination is a phenomenon that takes place much earlier than clinically detectable metastases. Notably, when comparing DNA alterations in the primary tumor and in CTC-c, 30–70% correspondence was observed. However, CTC-c were also characterized by exclusive alterations, suggesting either subclonality of the primary tumor or shedding from occult micrometastases [30].

The role of CTCs has also been investigated in lung cancer. Longitudinal changes in PD-L1 expression by CTCs were assessed in patients with advanced NSCLC treated with nivolumab. After 8 weeks of therapy, the ratio of PD-L1 positive CTCs was reported to be significantly lower than at baseline. PFS was, moreover, increased in patients with a higher PD-L1 positivity rate. This suggests that even though it may be important that the PD-L1 positivity rates after the beginning of the therapy become lower than the ones reported at baseline, it is preferable to have a few cancer cells that show a persistent response to the therapy. Furthermore, at 6 months from the beginning of the therapy, higher PD-L1 positivity rates were found to be predictive of the long-term efficacy of PD-1 blockade, in contrast with that which was observed in the early phase of treatment [31]. Moreover, CTCs collected from peripheral blood samples were employed to analyze protein expression and evaluate drug sensitivity, which was ultimately found to correspond with the clinical outcome [32].

Further, CTC detection is associated with earlier cancer metastasis and reduced survival outcomes in patients with early pancreatic cancer and with increased risk of death in patients with metastatic castration resistant prostate cancer [33,34].

Liquid biopsy comprises several technologies that can provide additional data on cancer biology, treatment resistance and survival outcome. The integration of its multiparametric features with tissue biopsy will ultimately lead to new opportunities for personalized medicine.
